# Micro-/Nano-Scales Direct Cell Behavior on Biomaterial Surfaces

**DOI:** 10.3390/molecules24010075

**Published:** 2018-12-26

**Authors:** Shuo Wang, Jingan Li, Zixiao Zhou, Sheng Zhou, Zhenqing Hu

**Affiliations:** School of Material Science and Engineering & Henan Key Laboratory of Advanced Magnesium Alloy & Key Laboratory of materials processing and mold technology (Ministry of Education), Zhengzhou University, Zhengzhou 450001, China; snowsunny24@gmail.com (S.W.); ZhouZixiao323@163.com (Z.Z.); zhousheng729@163.com (S.Z.); huzhenqing666@163.com (Z.H.)

**Keywords:** biomaterials surface, surface functionalization, micro/nano scales, cell behavior

## Abstract

Cells are the smallest living units of a human body’s structure and function, and their behaviors should not be ignored in human physiological and pathological metabolic activities. Each cell has a different scale, and presents distinct responses to specific scales: Vascular endothelial cells may obtain a normal function when regulated by the 25 µm strips, but de-function if the scale is removed; stem cells can rapidly proliferate on the 30 nm scales nanotubes surface, but stop proliferating when the scale is changed to 100 nm. Therefore, micro and nano scales play a crucial role in directing cell behaviors on biomaterials surface. In recent years, a series of biomaterials surface with micro and/or nano scales, such as micro-patterns, nanotubes and nanoparticles, have been developed to control the target cell behavior, and further enhance the surface biocompatibility. This contribution will introduce the related research, and review the advances in the micro/nano scales for biomaterials surface functionalization.

## 1. Introduction

One of the key strategies of tissue engineering is to understand how cells respond to external guidance signals from the surrounding microenvironment. In addition to biological and chemical signals (such as growth factors, hormones, and small chemicals), physical cues including topography and hardness are also considered to be important factors that influence cell behaviors [1,2,3]. Cells exist in complex microenvironments with extracellular matrix (ECM) as the main component. The ECM of various natural tissues such as bone [4], cartilage [5], nerves [6] or blood vessels [7] are composed of micro scale and nano scale topographic patterns. Recent studies have shown that topographical factors such as size, shape, and geometric alignment have strong influence on the adhesion, migration, arrangement, and differentiation of many cells [8,9,10,11,12,13,14,15]. The size of the terrain seems to play a crucial role in regulating cell behavior. Although the responses of the cells to the topography are diverse and closely related to cell type, all types of cells appear to be affected by size (e.g., width, spacing, and feature depth).

**Figure 1:**
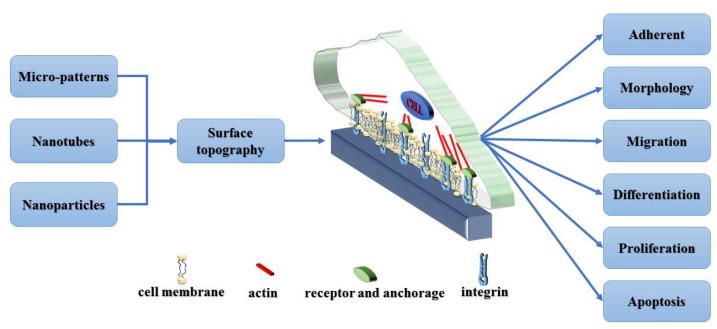
The schematic diagram of the effect of topographic scale on cell behavior.

The primary concern of this review is to emphasize the importance of different topographical scales, and their impact on related cellular behavior, including micro-patterns, nanotubes and nanoparticles (Figure 1). In order to develop materials and surfaces suitable for tissue engineering applications, it is important to understand how cells respond to topography at different length scales.

## 2. Application of Micro-Patterns in Biomaterials

**Figure 2:**
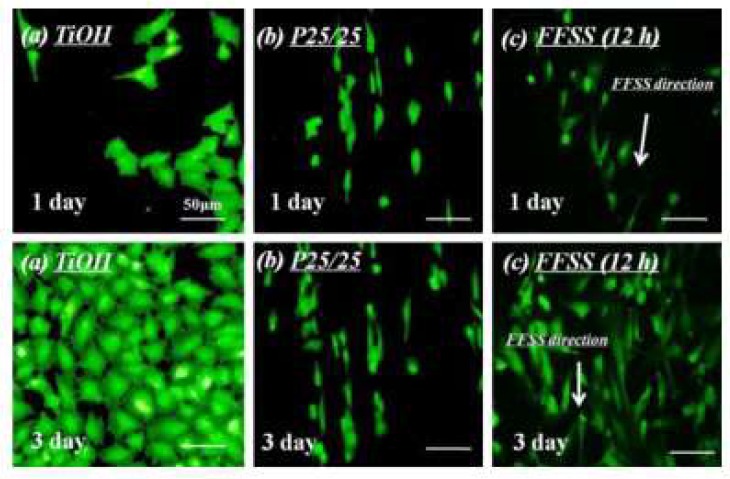
In the cited images, Li et al. prepared hyaluronic acid micro-strips with 25 µm size (samples were labeled as (**b**) P25/25) on the NaOH treated titanium surface to control the vascular endothelial cells’ morphology. The study demonstrated that the vascular endothelial cells controlled by P25/25 presented a similar morphology (elongation) with the vascular endothelial cells under 15 dyn/cm^2^ fluid flow shear stress (samples were labeled as (**c**) FFSS, it is also the average blood flow shear stress in the human blood vessels). The vascular endothelial cells on the single NaOH treated titanium control (samples were labeled as (**a**) TiOH, and it was flat) showed round and polygonal morphologies which were different from the cells on P25/25 or under FFSS [22]. Copyright 2013, Elsevier.

**Figure 3:**
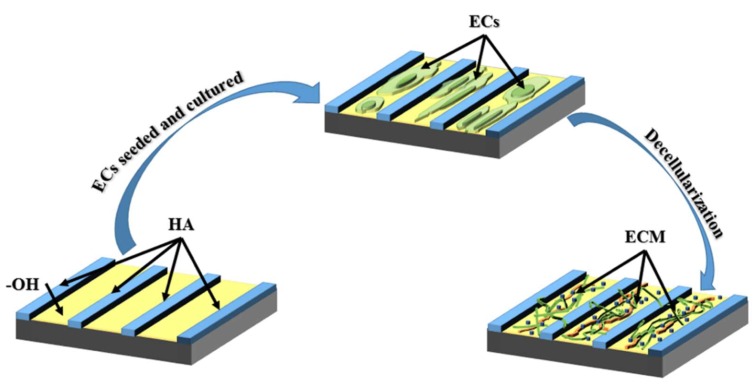
The scheme of preparing hyaluronic acid (HA)-patterned extracellular matrix (ECM): To obtain better biocompatibility, Li et al. controlled the vascular endothelial cells (EC) via preparing hyaluronic acid micro-pattern on materials surfaces to simulate the in vivo blood flow condition, and the extracellular matrix secreted by the patterned EC also showed similar property with the EC in vivo; Further investigation proved that the ECM left on the HA micro-pattern after decellularization had better biocompatibility compared to the ECM secreted by those EC on non-patterned surface [23].

Cell micro-patterns have a wide range of applications in tissue engineering. The activity of cells is highly dependent on their microenvironment, such as the scaffold and surrounding cells. Therefore, designing the cellular microenvironment is of great significance. There are several methods that allow the creation of micro scaled bio-patterns on materials, and most studies use lithography such as micro-contact printing (MCP) [16], microfluidic patterning [17], micromolding [18] or ultraviolet (UV) lithography [19]. Hirschbiel et al. used UV photolithography to form micro-patterns on polycarbonate (PC) films to regulate cell behavior [20]: Co-culture of PAC2 fibroblasts cells showed that irradiation was beneficial to cell adhesion and growth. In order to control the adhesion of MC3T3 cells, Chollet et al. used UV excimer laser ablation and photolithography to covalently graft arginine-glycine-aspartic acid (RGD)-containing peptides onto the polyethylene terephthalate (PET) surface to form micro-patterns [21]. The results showed that the RGD peptide enhanced the adhesion of the cells to PET, and the spreading of the cells appeared to be related to the width of the RGD layer, with the best effect at 25–125 μm. Regardless of the space between the RGD domains, cells appeared to be aligned and connected on the RGD region. In addition, the size of the pattern (the width of the RGD layer) also seemed to be a very important parameter, and the MC3T3 cell spread better when the RGD thickness was about 100 μm. To regulate the distribution, morphological, and cytokine secretion of human vascular endothelial cells (EC), Li et al. fabricated hyaluronic acid (HA) micro-strips with different sizes on the cardiovascular materials surface to simulate the blood flow effect on EC in vitro. Their series of biological evaluation indicated that the HA micro-strips of 25 µm obtained a similar effect as with blood flow on regulating endothelial cells (Figure 2) [22]. The differences in the distribution and morphology of EC on each differently-sized micro-strip indicate that the size of the micro-strip is closely related to its effect on EC regulation. Although the HA micro-strip on the materials surfaces can elongate the morphology of EC, thereby enhancing the deposition of endothelial ECM, the HA micro-strip showed poor blood compatibility [22]. In order to solve this problem, Li et al. then proposed a method for combining HA micro-strips with EC decellularization as shown in Figure 3 [23]. Their further biological evaluation consistently demonstrated HA-patterned endothelial ECM had series of functions to improve the surface biocompatibility [24]. Nevertheless, the above modification techniques may be limited to the continuous flat surface of large devices, because the micro scale on the small devices with good elasticity (such as stents) may change when the devices are opened.

Laser micro-patterning has been developed rapidly because of its rapid and precise processing [25]. Liang et al. prepared vascular smooth muscle cell (VSMC) micro/nano biomimetic surface patterns on 316L stainless steel (316L SS) by using femtosecond laser (FSL), processing to achieve the purpose of rapid endothelialization [26]. Cell and animal experiments show that it can promote the adhesion and proliferation of human umbilical vein endothelial cells (HUVECs) and inhibit the proliferation of VSMC as to achieve rapid endothelialization. In recent years, the appearance of direct laser writing (DLW) has made it possible to form micro/nano scale three-dimensional (3D) micro-patterns on the surface of materials [27,28]. Klein et al. prepared polymer scaffolds with 3D microstructures from two different photoresist by continuous DLW method, and achieved the purpose of regulating cell adhesion from 3D scale for the first time [29]. Richter et al. used DLW for the first time to control the chemical properties of the surface of 3D micro-scaffolds [30]. Claus et al. used two new photoresists with photo-reactive groups to realize the multiplex and simultaneous orthogonal functionalization of 3D microstructures by DLW technology for the first time, which has a good prospect of application [31]. On the basis of their original work, Richter et al. prepared a DLW 3D micro-scaffold by combining a protein repellent photoresist with a protein adhesive and a photo-activated passivated adhesive. The co-culture of epithelial cells (A549) showed that the bioactive functionalization of two different ECM proteins on the 3D micro-scaffold was realized for the first time [32].

Electro-spinning is also a common method in tissue engineering [33]. Both grooved micro-patterns and electro-spun fibers can affect the adhesion and proliferation of EC [34]. Electro-spinning techniques are commonly used to fabricate tissue-engineered fiber scaffolds [35]. Yoshimoto et al. made poly (e-caprolactone) (PCL) into nanofiber scaffolds by electro-spinning. Cell experiments showed that the scaffolds had potential for bone repair [36]. Min et al. also used silk fibroin (SF) to make wound dressings by electro-spinning [37]. Li et al. combined bone morphogenetic protein-2 (BMP-2) and nanoparticulate hydroxyapatite with electrostatic spinning scaffold for bone tissue engineering [38]. Frohbergh et al. enhanced the biocompatibility of the electro-spinning scaffolds by crosslinking with genipin [39]. Moffa et al. used electro-spinning and soft lithography to fabricate nanofiber scaffolds with different micro-patterns [40]. Through observation of various behaviors of EC, it is found that micro-patterns play a beneficial role in the regulation of EC and promote the regeneration of EC with complete functions. Rogers et al. combined the additive manufacturing (AM) method with electro-spinning to prepare fiber scaffolds with surface micro-patterns [41]. Observing the adhesion and proliferation of 3T3 fibroblasts, the cells first adhered to the micro-patterned regions and exhibited an undulating structure after proliferation in the sinusoidal region, indicating that the patterning of the electro-spun scaffold can affect the behavior of the cells.

In addition to EC and 3T3 cells, mesenchymal stem cells (MSCs) that are present around the blood vessels also play an important role in tissue engineering and bone repair [42]. Phipps et al. used polyethylene oxide (PEO) sacrificial fiber to enhance cell infiltration in PCL, collagen I and hydroxyapatite electro-spun scaffolds, and MSC adhesion [43]. To study the behavior of human bone marrow mesenchymal stem cells (HMSCs), Ynsa et al. used a high-energy proton beam to prepare a geometric micro-patterned surface in which silicon and porous silicon (Si/PSI) were combined [44]. The Si/PSI patterns promoted cell adhesion and migration and maintained the expression of major bone transcription factors, as well as intercellular interactions with good results. The surface morphology, charge density, surface energy and wettability of biological materials all affected the proliferation and differentiation of stem cells [45,46,47]. Tan et al. designed a micro-patterned polymer brush platform that could exclude the effects of other surface properties and study the effects of microenvironment on epidermal stem cell differentiation [48]. It was found that moderate hydrophobicity did not affect the differentiation and adhesion of stem cells, while strong negative surface potential increased the differentiation rate. This platform could simultaneously detect multiple cell microenvironment parameters, which had a certain impetus to the development of biomaterials.

Surface micro-patterning of biomaterials is an important mean of regulating cell behavior [49,50]. Changes in topographic and surface mechanical properties lead to changes in cell morphology and function [51,52,53]. Bulk metallic glass (BMGs) are especially suitable for micro/nano scale processing due to their unique superior properties [54,55,56,57,58]. Because of its excellent stability, BMGs is capable of forming thermoplastic forming (TPF) in a subcooled liquid region that is significantly below the melting temperature [59]. Wang et al. found a method for preparing graded micro/nano patterns of platinum-BMG (Pt-BMG) on the corresponding length scale [60]. The morphology of macrophages and fibroblasts was analyzed by in vitro cell culture experiments, and these two cell types were critical for the response to foreign bodies. The results showed that the morphological changes of macrophages and fibroblasts were significantly different, and the activation states were less. Different length scales could systematically influence the corresponding cell type-specific responses, and biomaterials with nano-topographic features could perform better and longer.

## 3. Application of Nanotubes in Biomaterials

In recent years, highly ordered, vertically-oriented titanium dioxide nanotube (TiO_2_ NT) arrays prepared by electrochemical anodization of titanium (Ti) and its alloys have attracted wide interest as biomedical coatings [61,62]. The diameter and length can be precisely determined by changing the anode parameters. Due to their self-organizing nature, even complexly shaped surfaces can be coated relatively easily [61]. TiO_2_ NT arrays have excellent biological properties in vivo and in vitro studies. For example, the TiO_2_ NT array has a good effect on the functions of various cells such as EC [63,64], vascular smooth muscle cells [63], human mesenchymal stem cells [65,66], and osteoblasts [67,68]. In order to improve the antibacterial properties, Gao et al. deposited Ti–Ag coating on Ti and then anodized to prepare silver oxide (Ag_2_O) nanoparticle embedded TiO_2_ NT array (NT–Ag_2_O array) [69]. Long-term culture results of *Staphylococcus aureus* and *Escherichia coli* showed that NT–Ag_2_O arrays have long-term effective antibacterial ability and can effectively kill adhering bacteria. The culture of MC3T3-E1 cells showed that NT–Ag_2_O arrays have good cytocompatibility and can promote the spreading, proliferation, and differentiation of osteoblasts. Wang et al. used a hybrid system of metal ion coordination polymers on the surface of titanium dioxide nanotubes (TNTs) to improve antibacterial properties [70]. They loaded antibacterial agents such as antibiotics and nano-silver particles into TNTs, and then sealed them with metal ions such as Zn^2+^ or Ag^+^ attachment coordination polymers (CPs). When pH is maintained at 7.4, the strong bonding of CPs can maintain the amount of released antimicrobial agents at a non-significant level. However, the acidic environment will trigger the coordination bond of the modified CP to open from the TNTs and release the antimicrobial agent. The proliferation of osteoblastic cells can be promoted by Zn^2+^, while the antibacterial capability can be enhanced by Ag^+^. Except zinc and silver, strontium (Sr) also has a dual role in improving bone formation and inhibiting bone resorption [71,72]. In order to incorporate Sr into TiO_2_ NTs, Chen et al. embedded TiO_2_ NTs containing Ag_2_O NPs (NT–Ag) into Ag_2_O nanoparticles (expressed as NT–Sr–Ag) by hydrothermal treatment in the Sr(OH)_2_ solution [73]. The morphology of the NTs was not altered by the hydrothermal treatment, but the amorphous TiO_2_ in NT–Ag converted into cubic SrTiO_3_. Sr^2+^ and Ag^+^ can be constantly released from NT–Sr–Ag, which possesses long-lasting antibacterial activity, good osteogenic, angiogenic activities and has clinical potential. Manganese (Mn) is also an element that is beneficial to bone regeneration [74]. Huang et al. used anodization and electrodeposition techniques to modify titanium implants with a two-layer coating co-doped with TNTs and hydroxyapatite with silver and manganese (AgMnHA) [75]. The addition of silver to hydroxyapatite improved its antibacterial properties, and the doping of manganese counteracted the potential cytotoxicity of the incorporated silver. The coating produced an excellent antibacterial effect by continuous release of silver ions. In vitro cell culture assays showed that osteoblast viability and alkaline phosphatase (ALP) activity were significantly improved. Therefore, the AgMnHA coating showed no significant cytotoxicity to osteoblasts and was able to produce sufficient osteoblast differentiation for osseointegration.

Bone tissue is a natural structure that includes both micro scale and nano scale [76]. For the sake of simulating the layered structure of bone tissue, Zhao et al. prepared biomimetic hierarchical micro/nano-textured surface topographies on Ti by acid etching and formed nano-tubular structures by anodization [77]. The micromorphology of acid etching induced higher initial cell adhesion and expression of osteogenic related genes, but other cellular behaviors such as proliferation, total intracellular protein synthesis and ALP activity, ECM deposition, and mineralization obviously decreased. After the addition of nanotubes to the surface of the micro-well, although cell adhesion and gene expression were slightly decreased, functions of cell proliferation, total intracellular protein synthesis and ALP activity, ECM deposition, and mineralization were able to be maintained or enhanced. Thus, better osseointegration in vivo may be resulted by this hierarchical micro/nano-textured surface topography.

Dopamine can be adhered to most materials and has good biocompatibility [78]. Jia et al. and Hong et al. used polydopamine (PDA) to load silver nanoparticles on titanium dioxide nanotubes (TiO_2_ NTs) to kill bacteria [79,80]. However, the polymerization of dopamine and the reaction of PDA with silver nitrate require longer reaction times. Furthermore, difficulty in controlling the deposition of silver nanoparticles may increase the risk of cytotoxicity [79,81,82]. To overcome these shortcomings, Ding et al. prepared the PDA film in a 90 °C water bath (PDA–H) by performing a rapid reaction on the TiO_2_ NTs [83]. In addition, the polydopamine–zinc (PDA–Zn) film formed on the surface of TiO_2_ NTs by dopamine and zinc nitrate solution significantly accelerated the reduction of Ag^+^ ions under the same heating conditions. The reaction time of the PDA–Zn film with silver nitrate was significantly shortened, and the silver nanoparticles deposited on the PDA–Zn film were more uniform than the silver nanoparticles on the PDA–H film. The test results showed that the PDA–Zn–Ag–TiO_2_ NTs material exhibited good antibacterial activity, lower cytotoxicity, and better biocompatibility.

More than TiO_2_ nanotubes, carbon nanotubes are also a commonly used material in tissue engineering [84]. Lately, natural polymers have provided more benefits than synthetic polymeric materials and have gained widespread interest in biomedical materials. Among these, bacterial cellulose (BC) has gained particular interest more recently [85,86]. BC is a polysaccharide used in the manufacture of reinforced paper, and has been studied as a medical material in recent years [87,88,89]. Gutierrez-Hernandez et al. combined natural BC with functionalized multi-walled carbon nanotubes (MWNTs) as raw biomaterials for 3D scaffolds for osteoblast culture [90], and functionalized MWNTs with native BC (secreted by *Gluconacetobacter xylinus*) to enhance the mechanical properties of BC. The results showed that the BC–MWNTs scaffold had a higher level of osteoblast activity, adhesion, and proliferation than the conventional culture substrates. These results indicate that the combination of BC and carbon nanomaterials has potential as a scaffold for bone regeneration.

## 4. Application of Nanoparticles in Biomaterials

Certain research demonstrated that the main cause of implant failure is the risk of biofilm-associated infection [91,92,93]. Scientists are designing new strategies to combat implant infections, focusing on the development of new biomaterials with anti-infective properties, and modifying the surface of biomaterials [94,95,96]. Recently, the application of silver nanoparticles (AgNPs) as antibacterial agents has aroused great interest [97,98,99,100,101]. However, nano-silver enters the cell and stays therein, resulting in local high concentration of silver ion distribution, causing clear toxicity and damage to cells, tissues and organs [102]. Therefore, maintaining the good antibacterial properties of nano-silver and reducing cytotoxicity is key to its application.

Because immobilizing AgNPs to limit their migration is critical to clinical success [102,103,104], Qin et al. prepared AgNPs in situ by silver plasma immersion ion implantation (PIII) and immobilized them on Ti surfaces [105]. The anti-biofilm activity of immobilized AgNPs was evaluated using the biofilm-producing strain *Staphylococcus ermidis*. Immobilized AgNPs reduced bacterial biofilm formation by inhibiting bacterial adhesion and *icaA/icaD* transcription. There was no obvious cytotoxicity in vitro. Anti-biofilm activity was independent of silver release of immobilized AgNPs, and they could protect multiple cycles of bacterial exposure in vitro and reduce implant-related prevention of periprosthetic infection (PPI) in vivo. The effectiveness depended on the Ag–PIII parameters, such as time. By changing the Ag–PIII parameter, the activity of the Ti anti-biofilm could be imparted, which indicates that the clinical use can be prolonged and safely used by reducing PPI. Taglietti et al. used the “layer-by-layer” (LBL) approach to modify the surface of the glass; after the aminosilylation of the glass and immersion in the AgNPs colloidal suspension, the self-assembled monolayer (SAM) of AgNPs immobilized on the glass surface was obtained [106]. The results showed that the new AgNPS modified glass had antibacterial/anti-biofilm ability, and not only exhibited good biological properties in terms of anti-infective properties, but also had valuable structural and physicochemical characteristics. More importantly, this surface modification method could be applied not only to glass, but also to most biological materials. Therefore, AgNPs were fixed on the surface to impart excellent antibacterial properties to the material.

Although Ti implants have good biocompatibility, the main challenge is still bacterial infection, with *Staphylococcus aureus* the main cause of orthopedic implant infection [107]. These bacteria form biofilms that are resistant to host defense mechanisms or antibiotics [108,109]. To solve this problem, Jin et al. used metal antibacterial agents such as silver and zinc to make the metal surface resistant to bacteria [110]. Recently, Mohandas et al. used a one-step hydrothermal method to prepare nano-titanium dioxide embedded in silver nanoparticles under alkaline pH conditions of silver salts [111]. Nano-silver-modified Ti surface not only had strong antibacterial properties, but also promoted the activity, proliferation, and osteogenic differentiation of MSCs. The incorporation of nano-silver into surface-modified Ti by a simple one-step hydrothermal method is a good method for preparing an implant having both antibacterial activity and cytocompatibility.

In the past few decades, Ti has been often used as a medical metal implant in orthopedics. However, as a bio-inert metal implant, it cannot induce bone regeneration after implantation. Bone morphogenetic protein-2 (BMP-2) is one of the important growth factors that induce bone marrow mesenchymal stem cell migration and osteoblast differentiation [112,113,114], while AgNPs have long-term effective antibacterial properties. In general, silver nanoparticles are prone to agglomeration, resulting in a large increase in local Ag concentration, which may affect cell activity. On the other hand, in order to maintain the activity of the growth factor, its fixation requires mild processing conditions. Therefore, Xie et al. used a combination of electrochemical deposition (ED) and electrostatic immobilization to prepare bone BMP-2 and hydroxyapatite-coated silver nanoparticles on the titanium surface, and selected chitosan (CS) as a stabilizer to achieve a uniform distribution of silver nanoparticles in the hydroxyapatite-coating [115]. While maintaining antibacterial activity, CS also reduced the toxicity of Ag. In addition, CS promoted the immobilization of BMP by electrostatic interactions between biomolecules, including CS, heparin, and BMP. In vitro and in vivo studies have shown that these coatings have high osteoinductivity and antibacterial properties. This method can also be used for surface modification of various metallic implants.

## 5. Conclusions

Specific scales are important parameters for determining the behavior of cells on the surface of biological materials. Cell adhesion, morphology, alignment, and contact guidance are strongly influenced by topographical features of series of micro/nano structures on the materials surfaces, and not only limited to micro-patterns, nanotubes and nanoparticles. In particular, there may be a specific range of scales that will most significantly improve these cellular behaviors. This optimal scale varies with cell type, substrate material and topography. In certain conditions, it seems that the reasonable combination of nano scale and micro scale topography may create better cell microenvironment. Therefore, information about the importance of terrain scales will help design appropriate materials and platforms in tissue engineering.
